# Targeting Toll-like Receptor 2: synthetic diacylated lipopeptides polarize equine macrophages towards a pro-inflammatory phenotype

**DOI:** 10.3389/fimmu.2026.1720816

**Published:** 2026-03-03

**Authors:** Chiara Grazia De Ciucis, Floriana Fruscione, Filippo Dell’Anno, Susanna Zinellu, Emanuela Giaconi, Simone Loi, Nicolò Columbano, Giulia Franzoni, Elisabetta Razzuoli

**Affiliations:** 1 Department Of Public Health, Experimental and Forensic Medicine, University of Pavia, Pavia, Italy; 2 National Reference Center of Veterinary and Comparative Oncology (CEROVEC), Istituto Zooprofilattico Sperimentale del Piemonte, Genova, Italy; 3 Department of Animal Health, Istituto Zooprofilattico Sperimentale della Sardegna, Sassari, Italy; 4 Department of Veterinary Medicine, University of Sassari, Sassari, Italy

**Keywords:** cytokines, gene expression, horse macrophages, macrophage polarization, TLR-2 agonist

## Abstract

Toll-like receptors (TLRs) are a group of pattern recognition receptors (PRRs), that play critical roles in initiating host immune defenses. TLR-2 agonists can activate innate immune cells and thus are attracting increasing attention as prophylactic and/or therapeutic agents against infectious diseases or in cancer immunotherapy. In this work, the impact of three synthetic diacylated lipopeptides (Mag-Pam2Cys_P48, MagPam2Cys_P80, and Mag-Pam2Cys_MAG1000) on equine monocyte-derived macrophages (moMΦ) phenotype and functionality was thoroughly investigated. MoMΦ were generated *in vitro* from circulating monocytes, and they were stimulated with these TLR-2 agonists, alongside untreated controls. The immunomodulatory effect was evaluated by RT-qPCR (expression of key immune genes) and ELISA multiplex (release of cytokines). Subsequently, the impact of MagPam2Cys_P80 on the phenotype of cells stimulated with IL-4 or IL-10 (‘M2-related’ cytokines) was investigated. We observed that stimulation with the three synthetic diacylated lipopeptides polarizes moMΦ towards a pro-inflammatory phenotype, with enhanced induction/release of pro-inflammatory cytokines, but with lower intensity compared to classical activation (IFN-γ + LPS). No differences between these agonists were detected, thus one of them (Mag-Pam2Cys_P80) was selected for further experiments with moM(IL-4) or moM(IL-10). Our data revealed that MagPam2Cys_P80 triggered increased release of IL-8, but not IL-1β, from moM(IL-10) 24 h after stimulation. In addition, TNF release was not observed when cells were simultaneously stimulated with IL-10. These data suggest that the inflammatory activity evoked by those agonist compounds could be partially mitigated *in vivo* by the release of anti-inflammatory molecules (e.g. IL-10), avoiding a potentially harmful dysregulated inflammatory response.

## Graphical Abstract

**Figure:**
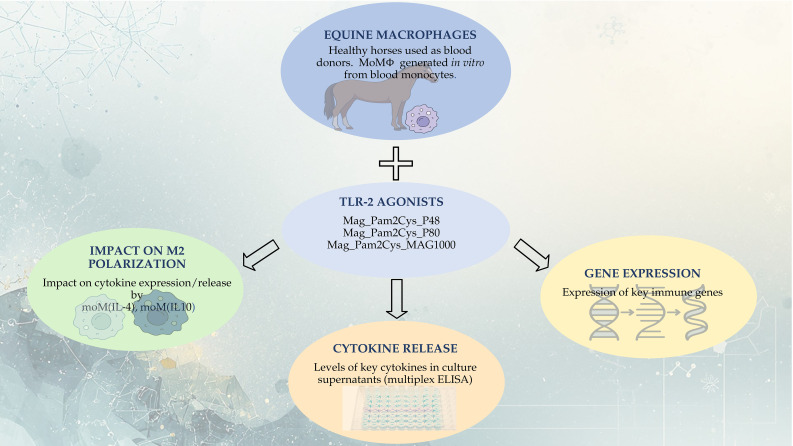

## 1 Introduction

Innate immunity is the first line of defense against invading pathogens, and a crucial role is played by pattern recognition receptors (PRRs), which recognize molecules expressed by pathogens, named pathogen-associated molecular patterns (PAMPs), and endogenous ligands (danger-associated molecular patterns or DAMPs). Toll-like receptors (TLRs) are a group of PRRs expressed by several immune cells and are located either on the cell membrane (TLR-1, -2, -4, -5, -6) or intracellularly (TLR-3, -7, -8, -9). Cell surface TLRs recognize microbial lipopolysaccharide, lipopeptides, glycolipids, flagellin (1, 2), whereas intracellular TLRs bind foreign nucleic acids (3). The binding of agonists to TLR activates specific intracellular signaling cascades that initiate host defense reactions (4, 5).

TLR-agonists are attracting increasing attention as prophylactic and/or therapeutic agents against infectious diseases (6, 7) or in cancer immunotherapy (8) due to their ability to activate immune cells. Among these, several TLR-2 agonists have shown promising immunomodulatory properties, including the lipopeptide macrophage-activating lipopeptide-2 (MALP-2) originated from *Mycoplasma fermentans*, which increased survival in mice infected with *Streptococcus pneumonia* (9, 10). Its synthetic analog S-[2,3-bis(palmitoyl oxy)propyl] cysteine (Pam2Cys) is a potent adjuvant, which has been incorporated into many vaccines (11–14) and increased protection against respiratory pathogens, such as influenza (12, 13). In animal models, prophylactic intranasal administration of a cell-surface TLR-2/6 agonist was effective against multiple respiratory viruses, including SARS-CoV-2, influenza, and rhinovirus (14–16). These agonists were able to activate innate immune defense pathways, in particular by stimulating nasal epithelial cells and recruited macrophages, leading to a reduction in viral load (17). Other TLR-2 agonists, such as Pam3Cys (synthetic triacylated lipoproteins), and SMP-105 (cell wall skeleton components), have been used in immunotherapy against bladder cancer (18). Bacillus Calmette-Guerin (BCG), a live attenuated *Mycobacterium bovis* derivative, possessed antitumor activity via TLR-2 and TLR-4 immune stimulation and was used in cancer treatment. Several studies reported that its injection frequently led to regressions of sarcoid, the most common equine cutaneous neoplasm, although adverse inflammatory reactions were frequently observed (19–21). A more recent paper reported that a TLR-2 agonist (acGM-1.8) polarized mice macrophages towards an anti-tumor phenotype and its injection in mice suppresses the growth of two tumor models (22).

So far, most of the studies on TLR-2 agonists were carried out in mice; however, the comparison between human, equine, and murine macrophages revealed functional differences (23–26), further emphasizing that rodents share fewer anatomical and immunological similarities with humans compared to large mammals, such as horses. Beyond their direct relevance in veterinary medicine, horses are attracting increasing attention as a large animal model in which to study macrophage biology and pathological processes shared with humans (26). Horses have been recognized as models for several human diseases, such as asthma, melanoma, metabolic syndrome, musculoskeletal diseases, vasculitis, squamous cell carcinoma and autoimmune uveitis (26–30). In addition, it was reported that equine alveolar macrophages (AMs) more closely resemble human rather than mice AMs in their response to LPS, suggesting that horses might represent a more suitable close-to human model for the study of macrophage associated lung-inflammation (25).

In this study, we investigated the effects of three synthetic diacylated lipopeptides (Mag-Pam2Cys_P48, Mag-Pam2Cys_P80, and Mag-Pam2Cys_MAG1000) on equine macrophages, which are indeed key players of the innate immune system and represent the first line of defense against invading pathogens (31). Macrophages are innate immune cells characterized by remarkable plasticity, as they can rapidly transform their phenotype and change their function in response to external stimuli (26, 32). The two antithetic extremes of polarized states are portrayed by classically activated (M1) macrophages, with high microbicidal or tumoricidal capacity, and alternatively activated (M2) macrophages, associated with immunosuppression and wound repair (32, 33). M1 macrophages can be generated *in vitro* by supplementation of culture media with IFN-γ and LPS, whereas IL-4, IL-10, TGF-β, or dexamethasone are regarded as ‘M2-related’ polarizing factors (32). Nevertheless, this exposure to diverse activators can lead to unique phenotypes; thus researchers have proposed a more accurate nomenclature, based on the activator/s used, such as M(IFN-γ + LPS), M(IL-4), M(IL-10), M(Mag-Pam2Cys_P48) (34).

We previously reported that the three TLR-2 agonists under investigation (Mag-Pam2Cys_P48, Mag-Pam2Cys_P80, and Mag-Pam2Cys_MAG1000) polarized porcine macrophages toward a pro-inflammatory and anti-microbial phenotype (35, 36) and in this study, we investigated whether these agonist compounds had similar effects on equine macrophages. Impact of these synthetic lipopeptides on the phenotype and functionality of equine macrophages was thoroughly analyzed using ELISA and gene expression assays, with the aim of laying the foundation for their *in vivo* evaluation as immunomodulators.

## 2 Materials and methods

### 2.1 Synthetic diacylated lipopeptides

Three diverse TLR-2 agonists S-[2–bis(palmitoyl)-propyl]cysteine (Pam2Cys) lipopeptides were used in this study: Mag-Pam2Cys_P48, Mag-Pam2Cys_P80 or Mag-Pam2Cys_Mag1000. These lipopeptides were chemically synthesized based on the 14 amino acids following the cysteine immediately downstream of the signal peptide of three *M. agalactiae* lipoproteins (P48: CGDKYFKETEVDGV; P80: CVDKDYEELGKDTK; and MAG_1000: CQNDEYQELDYKKW) (Espikem, Prato, Italy) (36).

### 2.2 Generation of equine monocyte-derived macrophages

Six healthy horses of either sex (mare and geldings), aged 4–8 years old, were used as blood donors in this study. Horses were considered healthy based on normal physiological variables (heart rate and respiration), absence of illness in the two months prior to sampling, and up-to-date vaccination and deworming status. The animals used in the study presented rectal temperature < 38.6 °C and did not receive any pharmacological treatment in the four weeks before bleeding.

For each animal, 30–45 mL of blood was collected using heparin as an anticoagulant (for peripheral blood mononuclear cells separation) and additionally 10–15 mL were collected in tubes without anticoagulant (for serum separation). Blood sampling was approved by the Ethics Committee of the Istituto Zooprofilattico Sperimentale del Piemonte, Liguria e Valle d’Aosta (IZS PLV 12/19; protocol no. 14047/19). Prior to sampling, informed consent was obtained from the horse owners.

First, whole blood without anticoagulant was centrifuged at 3000 g for 10 min, and serum was separated and placed in a clean tube. Then, horse peripheral blood mononuclear cells (PBMCs) were prepared by diluting heparinized blood in phosphate buffered saline (PBS) 1:1, layering it over 20 mL of Histopaque-1077 (Sigma), and centrifuging it at 700 x g for 30 min at room temperature (RT), without braking, in a rotating bucket centrifuge. PBMCs were aspirated from the plasma-Histopaque interface and washed three times in PBS by centrifugation at 1000 x g for 5 min at 4 °C (37). Then, PBMCs were resuspended in RPMI-1640 supplemented with autologous horse serum (20%), 100 U/mL penicillin, 100 µg/mL streptomycin (complete RPMI, cRPMI), and human M-CSF (hM-CSF) (Thermo Fisher Scientific, Waltham, MA, USA) (50 ng/mL) and plated in Petri dishes at 2 x 10^6^ live cells/ml. Cells were incubated for 7 days at 37 °C in 5% CO_2_, then non-adherent leukocytes were removed. Adherent moMΦ were subsequently detached by gentle scraping, centrifuged at 200 × g for 8 min. Cell were counted and viability was determined using a trypan blue and Countess Automated Cell Counter (Thermo Fisher Scientific). Cells were re-suspended in cRPMI supplemented with 10% autologous horse serum, and seeded in 24-well plates (Greiner CELLSTAR, Sigma-Aldrich, Saint Louis, MO, USA) (5 × 10^5^ live cells per well). After plating, cells were incubated for a further 24 h at 37 °C in 5% CO_2_ without hM-CSF, before stimulation (37, 38).

### 2.3 Stimulation of equine monocyte-derived macrophages

To evaluate the immunomodulatory impact of the three TLR-2 agonists under study, moMΦ from four different blood donor horses were left untreated (cRMPI only, moMΦ) or were treated with 100 ng/mL of the diverse TLR-2 agonists: Mag-Pam2Cys_P48, Mag-Pam2Cys_P80, Mag-Pam2Cys_MAG1000 (one single agonist per condition).

To evaluate the differences between classical activation and stimulation with a TLR-2 agonist, cells from four diverse blood donor horses were used. Cells were left untreated (cRMPI only, moMΦ) or were treated with 100 ng/mL of Mag-Pam2Cys_P80 or were classically activated (moM1). Classical activation was achieved by addition to culture media of both 100 ng/mL of recombinant equine IFN-γ (R&D, Minneapolis, MN, USA) and 100 ng/mL of LPS (Lipopolysaccharide from Escherichia coli 0111:B4; Sigma-Aldrich) for 24 h.

To evaluate the impact of a TLR-2 agonist on alternatively activated macrophages (moM2), cells from four diverse blood donor horses were used. Cells were left untreated (cRMPI only, moMΦ) or stimulated with 20 ng/mL of recombinant equine IL-4 (moM(IL-4)) or with 20 ng/mL of recombinant equine IL-10 (moM(IL-10) (both R&D) for 24 h. For all the three macrophage subsets (moMΦ, moM(IL-4), moM(IL-10)), two conditions were tested: one with and one without addition of 100 ng/mL of MagPam2Cys_P80.

### 2.4 Cytokine quantification

Culture supernatants were collected at 24 h post-stimulation, centrifuged (at 2500 × g for 3 min), and stored at −80 °C until analysis. The concentrations of GM-CSF, IL-1α, IL-1β, IL-6, IL-8, IL-10, IL-12p70, IL-18, and TNF were determined using the Equine Cytokine/Chemokine Magnetic Bead Panel Multiplex assay (Merck Millipore, Darmstadt, Germany) and a Bioplex MAGPIX Multiplex Reader (Bio-Rad, Hercules, CA, USA), following the manufacturer’s instructions. Experiments were performed in technical duplicates, as previously described (39).

### 2.5 RT-qPCR

Changes in the mRNA expression profiles were evaluated as previously described (36). Briefly, moMΦ were seeded in 24-well plates and either left untreated or stimulated as described in 2.3. After 3 h or 24 h, cells were lysed using buffer RTL (Qiagen, Hilden, Germany). Total RNA was extracted using the RNeasy Mini Kit, treated with the RNase-Free DNase Set (Qiagen, Milan, Italy), and eluted in 50 µL of ultrapure RNase-free water (Promega, Madison, WI, USA). Each RNA sample was quantified using a Qubit 3.0 fluorometer (Thermo Fisher Scientific) with the Qubit™ RNA HS Assay Kit (Invitrogen, Thermo Fisher Scientific (Carlsbad, CA, USA). 250 ng of purified RNA were used as a template for cDNA synthesis using the iScript cDNA Synthesis Kit (Bio-Rad, Hercules, CA, USA) according to the manufacturer’s instructions. RT-qPCR was subsequently to evaluate expression of several genes of the innate immune system: *Interleukin* (*IL*) 1*β*, *IL6*, *C-X-C Motif Chemokine Ligand 8* (*CXCL8*), *IL10*, *IL12A, IL12B*, *IL18*, *Interferon Beta* (*IFNB)*, *Transforming Growth Factor Beta 1* (*TGFB1*), *Nitric Oxide Synthase 2* (*NOS2)*, and *Nuclear Factor k B/p65* (*NFkB/p65*), Tumor Necrosis Factor (*TNF*), using the primer sets reported in **Supplementary Table S1**. Real-time PCR amplification was carried out on a CFX96™ Real-Time System with *Beta-2-Microglobulin* (*B2M*) and *Actin beta (ACTB*) used as reference genes.(For each sample, relative gene expression was calculated from the quantification cycle (Cq) values using the 2^−ΔΔCq^ method; RT-qPCR experiments were performed in technical duplicate, as previously described (36).

### 2.6 Statistical analysis

*In vitro* data were statistically analyzed with STATA 17 (StataCorp LLC, Texas 77845 USA) and graphically presented with GraphPad Prism 10.01 (GraphPad Software Inc., La Jolla, CA, USA).

To evaluate the impact of the diverse TLR-2 agonists and classical activation (IFN-γ + LPS) on moMΦ, a Kruskal-Wallis test was first performed to verify the presence of overall differences between groups. Subsequently, if significant, pairwise multiple comparisons were performed using the Dunn test. The Bonferroni correction was applied to control for Type I errors due to multiple comparisons. To evaluate the impact of a TLR-2 agonist on moM2, each gene/protein was evaluated within each experimental subset by pairwise comparison of moMΦ *vs* moM(MagCysPam_P80), moM(IL-4) *vs* moM(IL-4 + MagCysPam_P80), and moM(IL-10) *vs* moM(IL-10 + MagCysPam_P80) using the Mann–Whitney U test. As each comparison addressed a distinct biological hypothesis, no adjustment was applied across subsets.

All the analysis was conducted maintaining a statistical significance level set at p < 0.05, while tendencies were reported at p < 0.01.

## 3 Results

### 3.1 Immunomodulatory effects of three TLR-2 agonists

First, the immunomodulatory effects of three chemically synthesized TLR-2 agonists (MagPam2Cys_P48, MagPam2Cys_P80, and MagPam2Cys_MAG1000) was evaluated on equine moMΦ.

**Figure 1:**
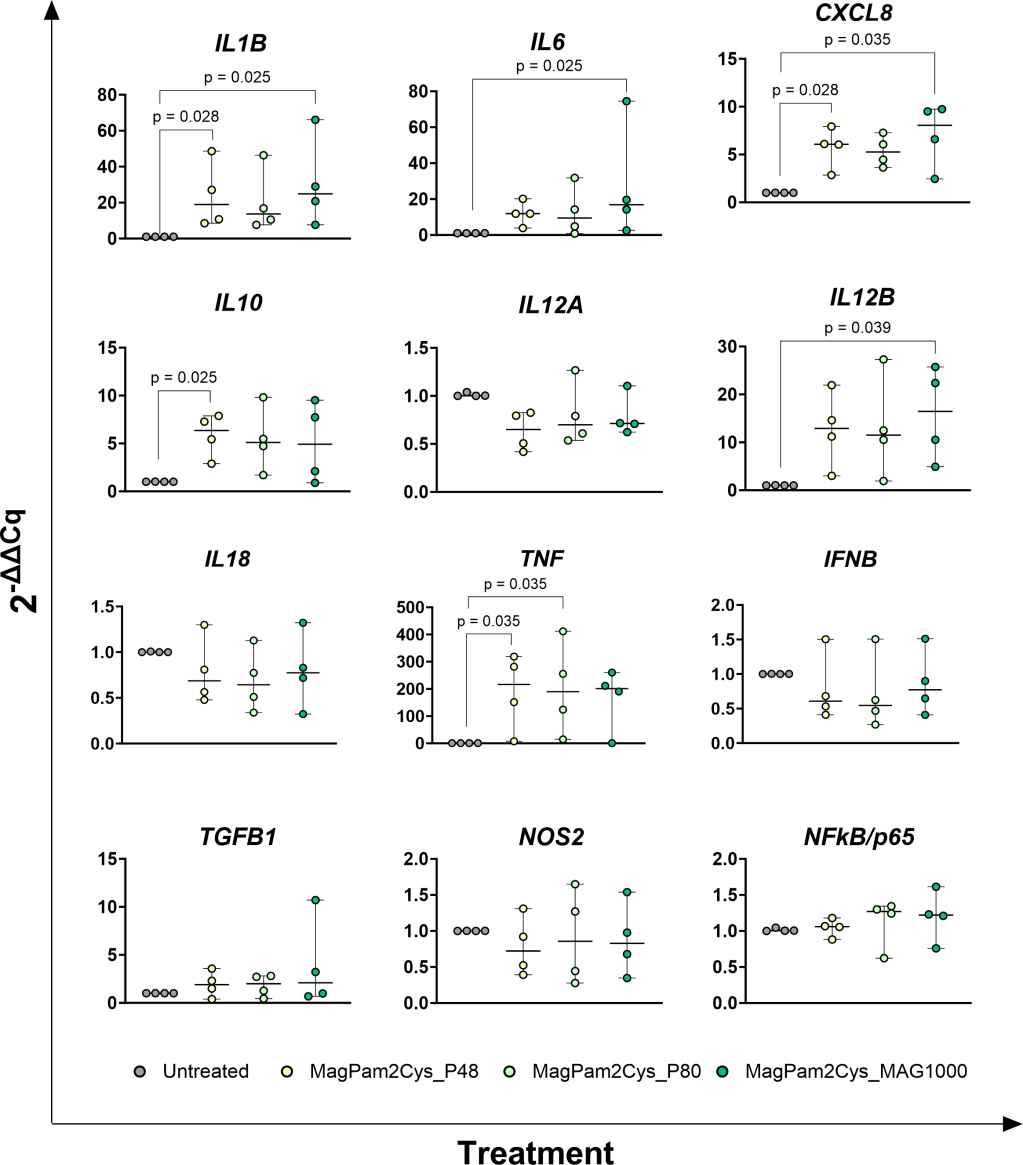
Impact of three diverse TLR-2 agonists on early expression (3 h) of key immune genes by equine macrophages. Equine moMΦ were left untreated or stimulated with three diverse TLR-2 agonists (MagPam2Cys_P48, MagPam2Cys_P80, MagPam2Cys_MAG1000), all at 100 ng/mL. 3 h later, cells were collected, and expressions of *IL1B*, *IL6*, *CXCL8*, *IL10*, *IL12A*, *IL12B*, *IL18, IFNB, TGFB1, iNOS, NFkB/p65* and *TNF* were determined through qRT-PCR. Data from four horses are presented. Differences between TLR-2-stimulated samples were compared to the corresponding untreated control (moMΦ) using a Kruskal–Wallis test followed by Dunn’s multiple comparison test. The Bonferroni correction was applied to control for Type I errors due to multiple comparisons; p value < 0.05 are displayed.

**Figure 2:**
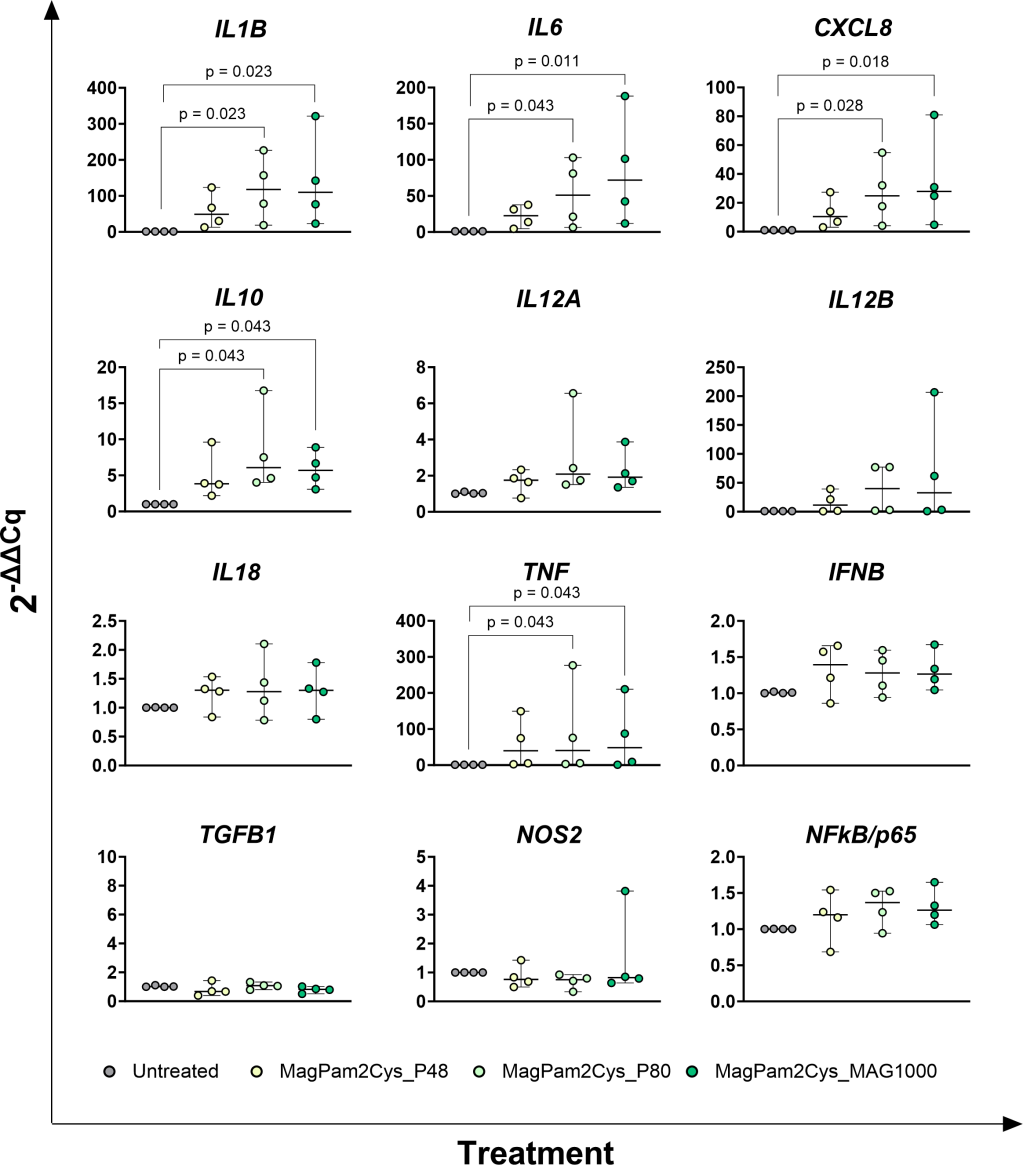
Impact of three diverse TLR-2 agonists on expression (24 h) of key immune genes by equine macrophages. Equine moMΦ were left untreated or stimulated with three diverse TLR-2 agonists (MagPam2Cys_P48, MagPam2Cys_P80, MagPam2Cys_MAG1000), all at 100 ng/mL. 24 h later, cells were collected, and expressions of *IL1B*, *IL6*, *CXCL8*, *IL10*, *IL12A*, *IL12B*, *IL18, IFNB, TGFB1, iNOS, NFkB/p65*and *TNF* were determined through qRT-PCR. Data from four horses are presented. Differences between TLR-2-stimulated samples were compared to the corresponding untreated control (moMΦ) using a Kruskal–Wallis test followed by Dunn’s multiple comparison test. The Bonferroni correction was applied to control for Type I errors due to multiple comparisons; p value < 0.05 are displayed.

**Figure 3:**
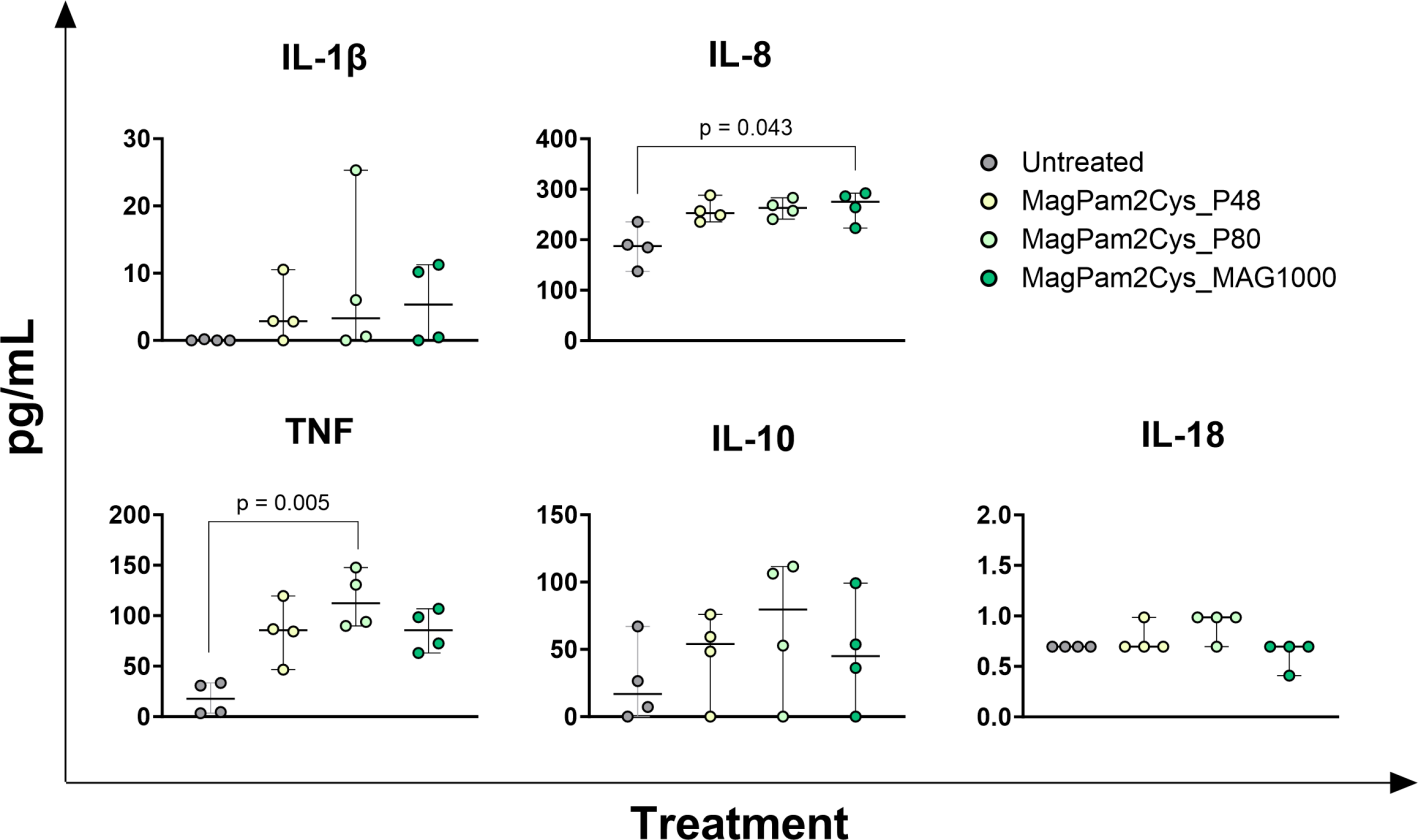
Impact of three diverse TLR-2 agonists on cytokine production by equine macrophages. Equine moMΦ were left untreated or stimulated with three diverse TLR-2 agonists (MagPam2Cys_P48, MagPam2Cys_P80, MagPam2Cys_MAG1000), all at 100 ng/mL. 24 h later, culture supernatants were collected, and concentrations of TNF, IL-1β, IL-8, IL-10, IL-18 were determined through multiplex ELISA. Data from four horses are presented. Differences between TLR-2-stimulated samples were compared to the corresponding untreated control (moMΦ) using a Kruskal–Wallis test followed by Dunn’s multiple comparison test. The Bonferroni correction was applied to control for Type I errors due to multiple comparisons; p value < 0.05 are displayed.

Changes in the expression of twelve key immune genes were analyzed by qRT-PCR. Early poststimulation (3 h) (**Figure 1**), we observed that MagPam2Cys_P48 and MagPam2Cys_MAG1000 triggered a significantly (p < 0.05) rise in the expression of the pro-inflammatory *IL1B*. MagPam2Cys_MAG1000 triggered a significantly (p < 0.05) increase for *IL6*, with a tendencies for MagPam2Cys_P48 (p = 0.077) and MagPam2Cys_P80 (p = 0.070). We observed a significantly (p < 0.05) increased expression also for the pro-inflammatory *CXCL8* and *TNF* when treated with MagPam2Cys_P48 and MagPam2Cys_MAG1000, and MagPam2Cys_P48 and MagPam2Cys_P80, respectively. A significantly (p < 0.05) rise in expression of the anti-inflammatory *IL10* was noted when treated with MagPam2Cys_P48; only a tendencies was observed for MagPam2Cys_P80 (p = 0.064) and MagPam2Cys_MAG1000 (p=0.094). Finally, the stimulation with MagPam2Cys_MAG1000 lead to an enhanced expression of *IL12B* (p < 0.05), and tendencies for MagPam2Cys_P48 (p = 0.052) and MagPam2Cys_P80 (p = 0.070) were noted (**Figure 1**). At 24 h post-stimulation (**Figure 2**), addition of MagPam2Cys_P80 and MagPam2Cys_Mag1000 to culture media resulted in a significantly (p < 0.05) increased expression of *IL1B, IL6*, *CXCL8*, *IL10* and *TNF*. At any tested time points (3 h and 24 h), no differences in expression of *IL12A, IL18, IFNB, TGFB1, NOS2* and *NFkB/p65* were observed between treated and untreated samples (**Figures 1**, **2**). 24 h post-stimulation, the concentrations of nine cytokines in culture supernatants were quantified by multiplex ELISA (**Figure 3**). A significantly release (p < 0.05) of pro-inflammatory cytokines TNF and IL-8 was observed when treated with MagPam2Cys_P80 and MagPam2Cys_MAG1000 respectively. MagPam2Cys_P80 induced a trend toward increased IL-8 release (p = 0.077). A trend was noted also for IL-1β and IL-10 release (p < 0.05). None of the tested TLR-2 agonists triggered in enhanced IL-18 concentration in culture supernatants. Concentrations of other two pro-inflammatory cytokines (IL-1α, IL-6), as well as IL-12p70 and GM-CSF, were below the assay detection limit. For these four cytokines, values lower of 24.8074 pg/mL (IL-1α), 1.7455 pg/mL (IL-6), 5.9235 pg/mL (IL-12p70), 4.8828 pg/mL (GM-CSF) were regarded as zero.

So far, our data revealed that all tested TLR-2 agonists polarized equine macrophages toward a pro-inflammatory phenotype.

### 3.2 Differences between classical activation and stimulation with a TLR-2 agonist

**Figure 4:**
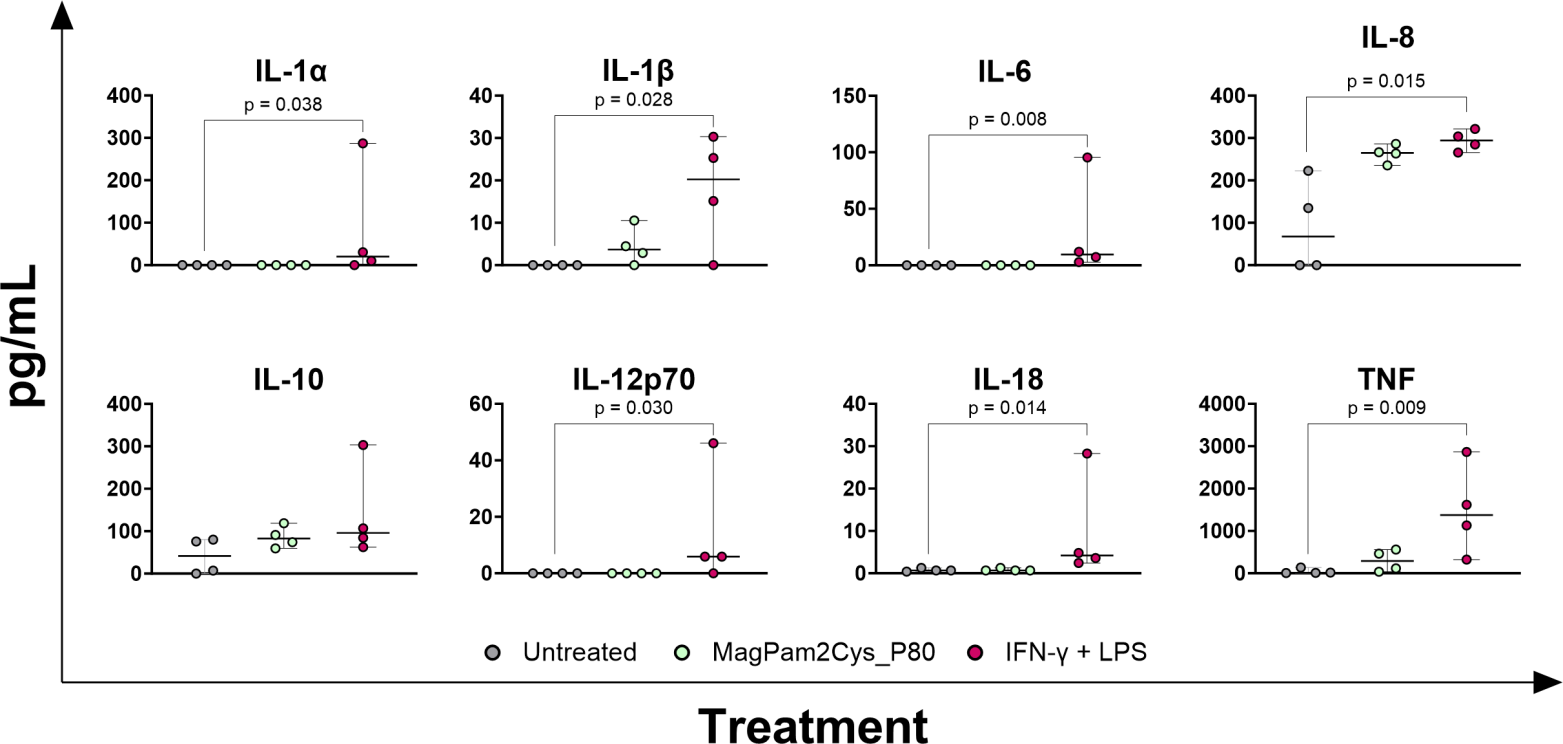
Cytokine release from equine moMΦ stimulated with a TLR-2 agonist or IFN-γ and LPS. Equine moMΦ were left stimulated with a TLR-2 agonist (MagPam2Cys_P80, 100 ng/mL) or with IFN-γ and LPS (classical activation). Untreated cells were used a control. 24 h later, culture supernatants were collected, and concentrations of IL-1α, IL-1β, IL-6, IL-8, IL-10, IL-12p70, IL-18, TNF were determined through multiplex ELISA. Data from four horses are presented. Differences between stimulated samples were compared to the corresponding untreated control (moMΦ) using a Kruskal–Wallis test followed by Dunn’s multiple comparison test. The Bonferroni correction was applied to control for Type I errors due to multiple comparisons; p value < 0.05 are displayed.

To this extent, we subsequently compared the impact of Mag-Pam2Cys_P80 with classical polarization (IFN-γ and LPS) by multiplex ELISA assay (**Figure 4**).

Our data revealed that stimulation with IFN-γ + LPS, but not Mag-Pam2Cys_P80, resulted in higher release (p < 0.05) of the pro-inflammatory IL-1α, IL-1β, IL-6, IL-8, IL-12p70, IL-18 and TNF compared to the untreated condition. Concentrations of GM-CSF were below the assay detection limit (4.8828 pg/mL).

### 3.3 Impact of TLR-2 agonist on M2 macrophages

We, then evaluated the effect of MagPam2Cys_P80 on the functionality of equine macrophages stimulated with two M2-related polarizing factors: IL-4 and IL-10. The impact of this TLR-2 agonist was investigated with both gene expression analysis and multiplex ELISA.

**Figure 5:**
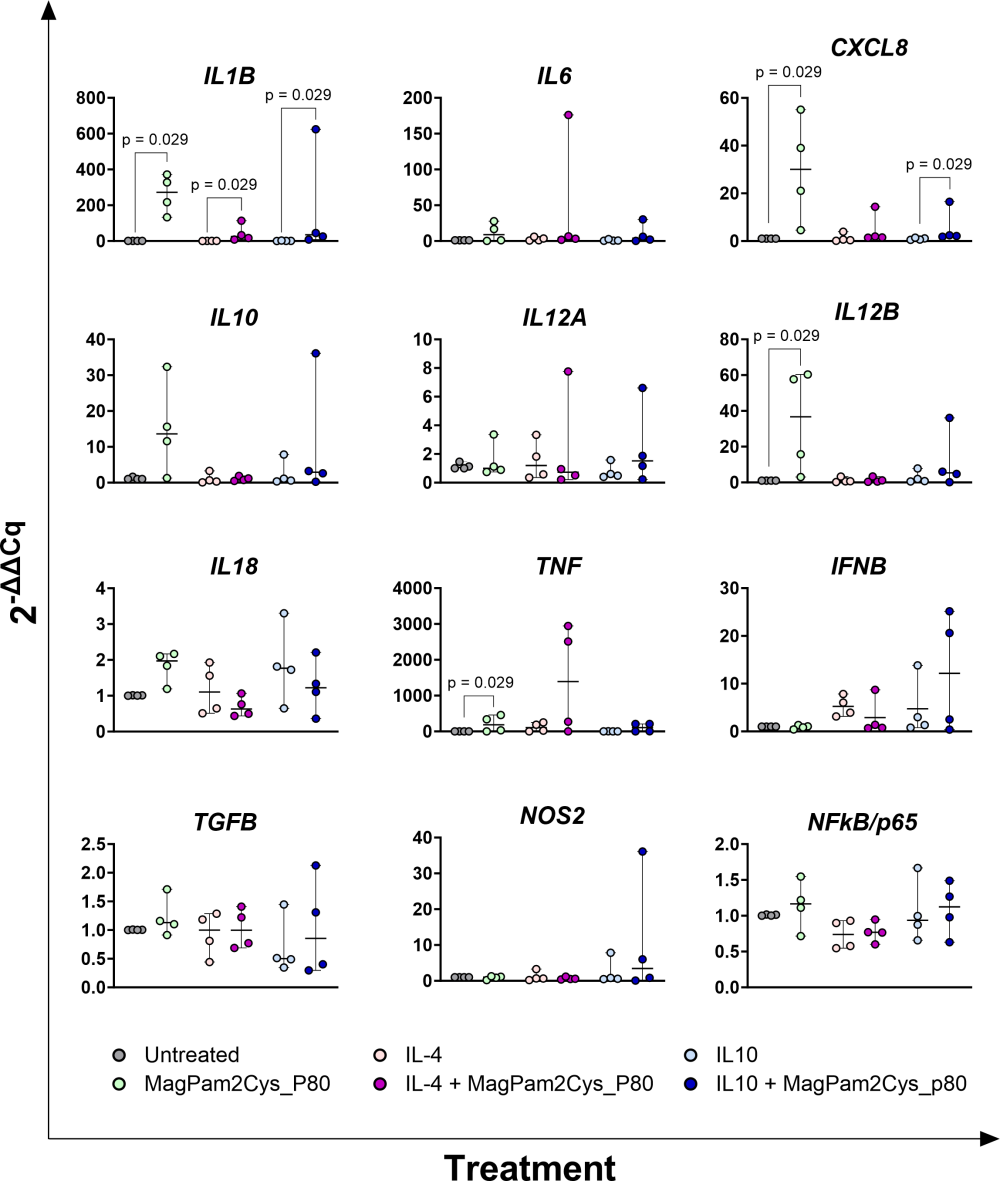
Impact of MagPam2Cys_P80 on expression of key immune genes by equine moMΦ stimulated with IL-4 or IL-10. Equine moMΦ were left untreated or stimulated with IL-4, IL-10. These macrophage subsets were simultaneously stimulated with a TLR-2 agonist (MagPam2Cys_P80) or left untreated. 24 h later, cells were collected, and expressions of *IL1B*, *IL6*, *CXCL8*, *IL10*, *IL12A*, *IL12B*, *IL18, IFNB, TGFB1, iNOS, NFkB/p65* and *TNF* were determined through qRT-PCR. Data from four horses are presented. For all the subsets, the impact of stimulation with MagPam2Cys_P80 was compared (untreated *vs* MagPam2Cys_P80; IL-4 *vs* IL-4 + MagPam2Cys_P80; IL-10 *vs* IL-10 + MagPam2Cys_P80) using a Mann-Whitney test; p value < 0.05 are displayed.

As displayed in **Figure 5**, addition of MagPam2Cys_P80 to culture media significantly (p < 0.05) raised gene expression of *IL1B, CXCL8*, *IL12B* and *TNF* compared to untreated moMФ. Moreover the addition of this TLR-2 agonist coupled with IL-10 resulted also in significant (p < 0.05) increased expression of *CXCL8* and *IL1B* compared to moM(IL-10). The addition of MagPam2Cys_P80 coupled with IL-4 showed a significant (p< 0.05) increased of *IL1B* expression compared to moM(IL-4). In all the subsets, expression of *IL6, IL10, IL12A, IL18, IFNB, TGFB1, NOS2* and *NFkB/p65* was not affected.

**Figure 6:**
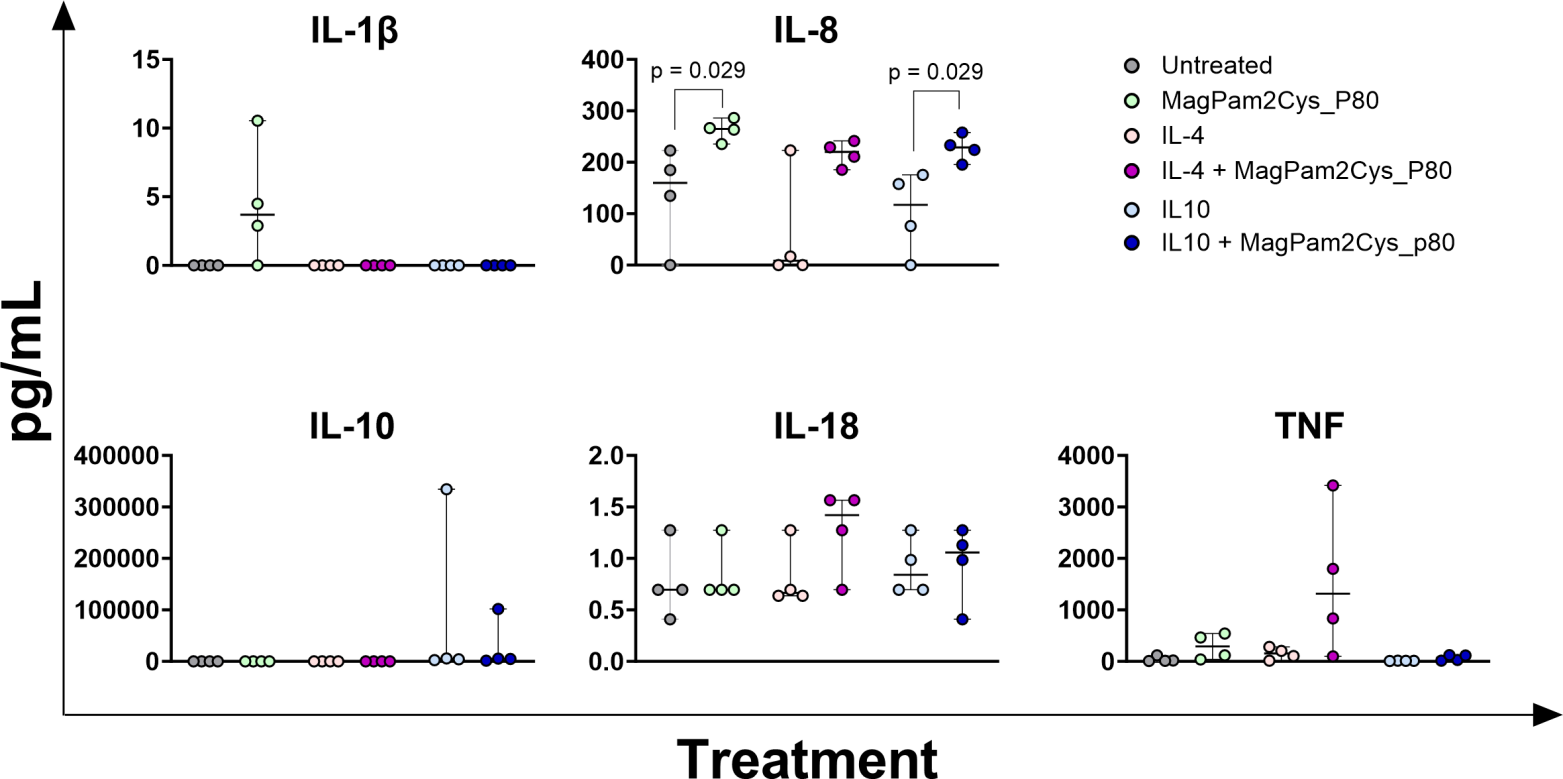
Impact of MagPam2Cys_P80 on cytokine release from equine moMΦ stimulated with IL-4 or IL-10. Equine moMΦ were left untreated or stimulated with IL-4, IL-10. These macrophage subsets were simultaneously stimulated with a TLR-2 agonist (MagPam2Cys_P80) or left untreated. 24 h later, culture supernatants were collected, and concentrations of IL-1β, IL-8, IL-10, IL-18, TNF were determined through multiplex ELISA. Data from four horses are presented. For all the subsets, the impact of stimulation with MagPam2Cys_P80 was compared (untreated *vs* MagPam2Cys_P80; IL-4 *vs* IL-4 + MagPam2Cys_P80; IL-10 *vs* IL-10 + MagPam2Cys_P80) using a Mann-Whitney test; p value < 0.05 are displayed.

Concentrations of key immune cytokines in culture supernatant were determined by multiplex ELISA. As presented in **Figure 6**, the addition of MagPam2Cys_P80 to culture media triggered a significantly (p < 0.05) release of IL-8 compared to untreated moMФ, similarly to the addition of MagPam2Cys_P80 coupled with IL-10 compared to moM(IL-10), but not in moM(IL-4) subset. Stimulation with MagPam2Cys_P80 resulted in an increased trend toward TNF release compared to untreated moMΦ (p = 0.057) but this was not observed in moM(IL-4) or moM(IL-10) subsets. IL-4 stimulation resulted in a slightly higher release of TNF from equine macrophages, which was further enhanced by MagPam2Cys_P80, although the differences in TNF concentrations between IL-4 *vs* IL-4 + MagPam2Cys_P80 were not statistically different, likely due to the variability between horses. MagPam2Cys_P80 induced a trend toward increased IL-1β release compared to untreated moMΦ, although this did not reach statistical significance. We also investigated the impact on the release of anti-inflammatory IL-10. In equine moMΦ, we observed that stimulation with IL-4 did not trigger release of this interleukin, and that was not affected by the addition of a TLR-2 agonist. Stimulation with IL-10 resulted in increased concentrations of IL-10 in culture supernatants, with no statistically significant differences between MagPam2Cys_P80-treated and moM(IL-10).

No differences were observed for IL-18 and amounts of IL-1α, IL-6, IL-12p70, and GM-CSF were below the assay detection limit. For these four cytokines, values lower of 24.8074 pg/mL (IL-1α), 1.7455 pg/mL (IL-6), 5.9235 pg/mL (IL-12p70), 4.8828 pg/mL (GM-CSF) were regarded as zero.

Our data revealed that stimulation with MagPam2Cys_P80 significantly affect the expression (*IL1B* and *CXCL8*) and release of pro-inflammatory cytokines (IL-8) from moM(IL-10). Concerning moM(IL-4) we demonstrated only a significant modulation of *IL1B* gene expression.

## 4 Discussion

TLR-2 agonists can activate innate immune cells and thus are attracting increasing attention as prophylactic and/or therapeutic agents against infectious diseases or in cancer immunotherapy. In this work, the immunomodulatory impact of three synthetic diacylated lipopeptides (Mag-Pam2Cys_P48, MagPam2Cys_P80, and Mag-Pam2Cys_MAG1000) on equine macrophages was investigated thoroughly. We observed that these synthetic lipopeptides polarize equine macrophages toward a pro-inflammatory phenotype, defined by enhanced induction and release of pro-inflammatory cytokines. These data might be a sign that *in vivo* these lipopeptides could trigger the release of IL-1β, TNF, and chemokines, such as IL-8, promoting the recruitment of immune cells to the site of injection and strengthening horses’ defense against invading pathogens, as observed in other *in vivo* experiments in mice or ferrets (14, 15, 40). In detail, Tan and collaborators reported that intranasal administration of a TLR-2 agonist triggered a cascade of inflammatory and innate immune signals, with recruitment of neutrophils and macrophages *in situ*, release of pro-inflammatory cytokines and IFN-γ, that resulted in increased resistance against challenge infection with a virulent influenza A virus (14). In another study, Deliyannis and colleagues observed that intranasal administration of the TLR-2 agonist INNA-X triggered rapid innate immune responses in nasal turbinates. Researchers observed that nasal epithelial cells and recruitment of macrophages worked together to limit the progression of the influenza virus to the lungs (17). Likewise, Proud and colleagues observed that intranasal administration of INNA-X in a ferret model increased resistance against SARS-CoV-2, reducing levels of viral RNA in the upper respiratory tract of immunized animals (15). Similarly, intranasal administration of the three TLR-2 agonists under investigation (Mag-Pam2Cys_P48, Mag-Pam2Cys_P80, and Mag-Pam2Cys_MAG1000) might increase resistance against respiratory pathogens in horses. Concerning *NOS2* expression, which encodes inducible nitric oxide synthase (iNOS), responsible for generating nitric oxide (NO) from arginine (41) as part of the host defense against pathogens, stimulation with TLR-2 agonists did not significantly modulate *NOS2* expression. This is consistent with previous studies reported that NO synthesis did not occur in equine macrophages following stimulation with IFN-γ and/or LPS (25, 33), because other pathways were activated (25), and this might occur also in response to our TLR-2 agonists.

Polarization toward an M1-like phenotype can be useful not only to enhance defense against intracellular pathogens, but also in the fight against malignancies (42). Tumor progression is associated with a change of macrophage phenotype and function toward a pro-tumor phenotype, defined by anti-inflammatory and pro-angiogenetic activities, which boost tumor growth and metastasis (43). An encouraging strategy in cancer immunotherapy is to reprogram tumor-associated macrophages (TAM), skewing their phenotype from a pro-tumor to an anti-tumor state (M1) (43). Feng and collaborators reported that a TLR-2 agonist (acGM-1.8) polarized mice macrophages towards an anti-tumor phenotype; *in vivo* experiments using a mouse model showed that its injection suppresses the growth of two tumor models (22). BCG is recognized by macrophages via TLR-2 and TLR-4 and triggers their polarization toward an M1 phenotype, characterized by secretion of pro-inflammatory cytokines, especially TNF, and ROS, with subsequent cytotoxic effect on sarcoid fibroblast and promoting the development of a pro-inflammatory tumor microenvironment (21). In our *in vitro* study, we observed that these TLR-2 agonists skew equine macrophage phenotype toward an anti-tumoral state. Furthermore, equine macrophages stimulated with IL-4 or IL-10 increased expression of *IL1B* in response to MagPam2Cys_P80 stimulation and were still able to release IL-8. Our data suggested that our TLR-2 agonist was able to partially shift equine macrophages toward an anti-tumor state, with the release of the cytokines, which *in vivo* should result in enhanced recruitment of immune cells to hopefully fight tumoral cells.

Overall, these molecules are indeed able to polarize equine macrophages toward a pro-inflammatory phenotype, more efficient in the fight against invading pathogens and tumors. Nevertheless, the inflammatory response should be tightly controlled to prevent potentially pathological overreaction to stressors (44). We observed that Mag-Pam2Cys_P80 polarized equine macrophages toward a pro-inflammatory phenotype, but moM1 released higher levels of pro-inflammatory IL-6, IL-12p70, IL-18, TNF compared to MagPam2Cys_P80, suggesting that this synthetic lipopeptide triggered a weaker pro-inflammatory response compared to classical activation, where cells are stimulated with both IFN-γ and LPS. Most importantly, concomitant administration of the anti-inflammatory IL-10 inhibited the expression and release of TNF in response to MagPam2Cys_P80, which is promising for the potential use of this TLR-2 agonist as an immunotherapeutic agent *in vivo*. IL-10 is indeed a potent anti-inflammatory cytokine, which is released to dampen the inflammatory response and to prevent inflammatory and autoimmune pathologies (45), and our data suggest that *in vivo* IL-10 can reduce the inflammatory response triggered by MagPam2Cys_P80.

Although this study present limitations, such as the small number of tested animals and the small number of tested time post-stimulation (3–24 h), these preliminary *in vitro* data are in favor of a potential application of these synthetic lipopeptides in the biomedical field: they polarized equine macrophages toward a pro-inflammatory phenotype, but this activation seemed to be controlled, to avoid pathological over-reaction to stressors.

Future studies should investigate the impact of these agonist compounds in later time points, such as 48 and 72 h post-stimulation to better predict their impact on macrophage phenotype and functionality at later stages. Another limitation of the study is that we investigated only moMΦ, whose response might differ from those of resident macrophages, such as AMs or peritoneal macrophages. AMs play a crucial role in the defense against pathogens in the equine airway, such as equine arteritis virus, equine influenza, equine herpesvirus 2, and opportunistic bacteria (26). It was reported that moMΦ and AMs responded differently to external stimuli (46) and these cells might evoke a diverse cytokine response following stimulation with these TLR-2 agonists.

Functional assays should also be performed, such as evaluating whether stimulation of macrophages (moMΦ and AMs) with these TLR-2 agonists limit the replication of respiratory pathogens, such as equine arteritis virus or equine influenza. As above stated, TLR-2 agonists are attracting attention not only to enhance defense against intracellular pathogens, but also in the fight against malignancies. Functional assays should be evaluated not only to check whether these agonists compounds are able to reprogram TAM toward an M1 phenotype, but also to reduce the growth of malignant cells, such as sarcoid-derived fibroblasts.

Overall, these preliminary *in vitro* data hint at a potential application of these synthetic lipopeptides in the biomedical field, but future *in vitro* and *vivo* studies should be performed to evaluate the use of these TLR-2 agonists as immunomodulators for horses.

## Data Availability

The raw data supporting the conclusions of this article will be made available by the authors, without undue reservation.
