# Supplementary material for: Targeting Toll-like Receptor 2: synthetic diacylated lipopeptides polarize equine macrophages towards a pro-inflammatory phenotype
Source: Front Immunol. 2026 Mar 3;17:1720816. doi: 10.3389/fimmu.2026.1720816 (PMC12991996; doi:10.3389/fimmu.2026.1720816)
Supplement: Supplementary file 1 [file Table1.docx]

**Supplementary Table S1. Primer set sequences for target equine genes**

| **Gene** | **Sequence** | **Accession Number** | **bp** |
| --- | --- | --- | --- |
| *IL1B* | For: 5’-TGATGCAGCTGTGCATTCAGT-3’  Rev: 5’-GCACAAAGCTCATGCAGAACA-3’ | NM_001082526.2 | 145 |
| *IL6* | For: 5’-TCAAGGGTGAAAAGGAAAACATC-3’  Rev: 5’-GGTGGTTACTTCTGGATTCTTC-3’ | NM_001082496.2 | 97 |
| *CXCL8* | For: 5’-CTGGCTGTGGCTCTCTTG-3’  Rev: 5’-CAGTTTGGGATTGAAAGGTTTG-3’ | [NM_001083951.2](https://www.ncbi.nlm.nih.gov/nucleotide/NM_001083951.2?report=genbank&log$=nuclalign&blast_rank=29&RID=D57H660S015) | 131 |
| *IL10* | For: 5’-TTCAGCAGGGTGAAGACTTTCT-3’  Rev: 5’-AAGGCTTGGCAACCCAGGTA-3’ | NM_001082490.3 | 106 |
| *IL12A* | For: 5’-CTGAGGACCGTCAGCAACAC-3’  Rev: 5’-GTTCGGGGCGAGTTCCAG-3’ | [NM_001082511.2](https://www.ncbi.nlm.nih.gov/nucleotide/NM_001082511.2?report=genbank&log$=nuclalign&blast_rank=6&RID=D58E8NCZ015) | 146 |
| *IL12B* | For: 5’-GATCGTGGTGGATGCTGTTC-3’  Rev: 5’-TCCACCTGCCGAGAATTCTT-3’ | NM_001082516.3 | 131 |
| *IL18* | For: 5’-AGCGGTAACCATCTCTGTGAA-3’  Rev: 5’-TCATGTCCTGGAACACTTCTCTG-3’ | NM_001082512.1 | 147 |
| *IFNB* | For: 5’-AAATACTACGGAAGGATCTCGCA-3’  Rev: 5’-AAGTTCCTGAGCATTTCCGCT-3’ | NM_001099440.1 | 91 |
| *TGFB1* | For: 5’-CGGAATGGCTGTCCTTTGATG-3’  Rev: 5’-CCCACGCGGAGTGTGTTAT-3’ | NM_001081849.1 | 126 |
| *NOS2* | For: 5’-CTTCTACCTCACGCTATCCAAT-3’  Rev: 5’-CTCCTTTGTTACGGCTTCCAG-3’ | NM_001081769.3 | 101 |
| *NFkB/p65* | For: 5’-GAGCCCATGGAGTTCCAGTA-3’  Rev: 5’-AGGTCTCATATGTCCTTTTGCGT-3’ | XM_023654462.2 | 81 |
| *TNF* | For: 5’-AGCCTCTTCTCCTTCCTCCTT-3’  Rev: 5’- CAGAGGGTTGATTGACTGGAA-3’ | NM_001081819.2 | 122 |
| *B2M* | For: 5’-GGCTACTCTCCCTGACTGG-3’  Rev:5’-TCAATCTCAGGCGGATGGAA-3’ | NM_001082502.3 | 135 |
| *ACTB* | For: 5’-CCCTGGAGAAGAGCTACGAG -3’  Rev:5’-CACAGGATTCCATGCCCAGG -3’ | NM_001081838.1 | 114 |
